# Case report: A rare case of skin abscess caused by coinfection of *Actinobaculum schaalii* and *Actinomyces turicensis*

**DOI:** 10.3389/fcimb.2024.1378197

**Published:** 2024-03-27

**Authors:** Peng Liu, Kangchao Sun, Rongguo Li, Xiaodi Chen

**Affiliations:** Department of Clinical Laboratory, Jinan Maternity and Child Care Hospital Affiliated to Shandong First Medical University, Jinan, China

**Keywords:** skin abscess, *Actinobaculum schaalii*, *Actinomyces turicensis*, coinfection, case report

## Abstract

Skin abscess is one of the most common infections of the skin and soft tissues. However, anaerobic bacteria are infrequently identified as the causative agents of this particular form of abscess. In this case, a 34-year-old pregnant woman was diagnosed with a skin abscess with the use of ultrasonography. The microbiological analysis results of the purulent fluid revealed the coinfection of *Actinobaculum schaalii* and *Actinomyces turicensis*. The patient was first treated empirically with 3 days of cefathiamidine, which resulted in no symptom improvement. Subsequently, a surgical procedure involving incision and draining was performed, with the administration of ceftriaxone. After 7 days of antibiotic intervention, the patient exhibited a satisfactory recovery. Clinicians need to be aware of other types of infections that might be attributed to *Actinobaculum schaalii* and *Actinomyces turicensis*, in addition to urinary tract infections.

## 1 Introduction

Skin abscess is one of the most common infections of the skin and soft tissues, and its incidence has exhibited an upward trend in recent years (Talan et al., 2016). The main clinical manifestations and signs of a skin abscess are redness, heat, swelling, pain, and impaired functionality (Baiu and Melendez, 2018). Bacterial infections, particularly infections caused by *Staphylococcus aureus*, are the predominant etiology of this type of abscess (Talan et al., 2016). However, recent studies have also indicated that skin abscesses can also be caused by anaerobic or facultative anaerobic bacteria (Accetto and Avguštin, 2021; Mah et al., 2022).

*Actinotignum schaalii*, initially described in 1997, is a small, rod-shaped, nonmotile, non–spore-forming, non–acid-fast, Gram-positive bacillus that is a typical component of the mucosa, skin, and urogenital tract microbiota (Hemenway et al., 2018; Kakodkar and Hamula, 2022). This organism exhibits anaerobic or facultative anaerobic characteristics, displaying optimal growth in anaerobic environments, limited growth in an atmosphere containing 5% CO_2_, and an inability to develop in aerobic circumstances (Yassin et al., 2017). Therefore, it is easily overlooked because of its special growth requirements. Recently, the utilization of advanced equipment within clinical microbiology laboratories has led to increasing recognition of *A. schaalii* as an opportunistic pathogen that is commonly associated with urinary tract infections, bloodstream infections, and Fournier’s gangrene (Yassin et al., 2017; Mah et al., 2022). However, abscess formation caused by *A. schaalii* is still rare (Panganiban and Gupta, 2020).

*Actinomyces turicensis*, which was first identified in 1995 through the utilization of 16S rRNA gene sequencing, is characterized as an anaerobic, Gram-positive bacterium (Agrafiotis and Lardinois, 2021). This microorganism belongs to the *Actinomyces* genus, which is known to be a constituent of the indigenous microbial community found in the mouth cavity, gastrointestinal system, and urogenital tract (Könönen and Wade, 2015; Wolff et al., 2022). Following mucosal erosion, *A. turicensis* has the potential to penetrate and induce pathogenicity, resulting in the emergence of an endogenous infection: actinomycosis. Actinomycosis is regarded as a chronic condition that commonly results in the development of granulomatous abscesses accompanied by the presence of purulent discharge (Boot et al., 2023). Consequently, this infection can result in necrosis, fibrosis, and the formation of adhesions with neighboring tissues or the development of draining sinuses (Wong et al., 2011).

Here, we describe a case of a skin abscess at the top of the crease of the buttocks without any apparent cause in a pregnant woman. The microbiological analysis results of the purulent fluid revealed the coinfection of *A. schaalii* and *A. turicensis*. To the best of our knowledge, this is the first report of a skin abscess caused by coinfection of these two bacteria.

## 2 Case report

### 2.1 Case description

**Figure 1:**
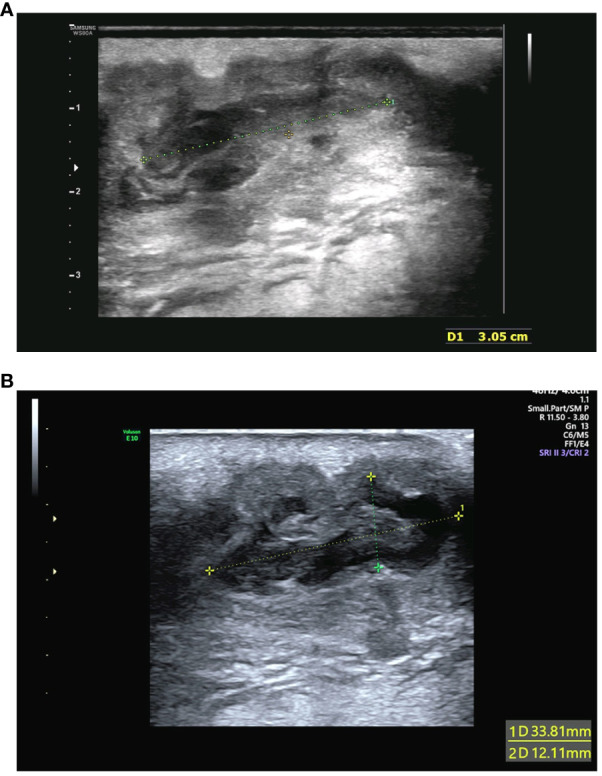
**(A)** Color flow Doppler ultrasonography of the abscess performed initially. The examination detected hypoechoic sound and identified a liquid dark area with poorly defined borders within the mass of approximately 31 mm × 11 mm in size. **(B)** Reexamination using color flow Doppler ultrasound after 3 days. The reexamination detected hypoechoic sound and revealed that the liquid dark area with poorly defined borders was 33 mm × 17 mm × 12 mm. The overall mass was approximately 50 mm × 35 mm × 25 mm in size.

A 34-year-old woman visited our hospital during week 18^+5^ of her pregnancy because of a sacrococcygeal mass at the top of the crease of the buttocks without any apparent cause. The skin was red and swollen, showing tenderness and fluctuating sensation. The patient had no fever and had blood pressure of 97/70 mmHg, heart rate 90 beats/min, body weight 64.7 kg, white blood cell count 14.01 × 10^9^ cells/L, neutrophil count 11.24 × 10^9^ cells/L, and C-reactive protein of 88.93 mg/L. The overall mass was approximately 60 mm × 23 mm and located entirely under the skin. Color flow Doppler ultrasonography of the abscess revealed a liquid dark area within the larger mass of approximately 31 mm × 11 mm in size (**Figure 1A**). A puncture was performed to collect samples inside the abscess for further culture. Anti-inflammatory treatment was administered empirically by intravenous injection of cefathiamidine (2 g once daily for three consecutive days). The patient was followed up three days after the last anti-inflammatory treatment. The abscess was slightly bigger than before, and the patient-assessed outcomes were poor. Reexamination using color flow Doppler ultrasound showed that the liquid dark area was 33 mm × 17 mm × 12 mm, and the overall mass was approximately 50 mm × 35 mm × 25 mm in size, suggesting a purulent inflammatory mass (**Figure 1B**). Abscess incision and drainage were performed. During surgery, 20 mL purulent fluid was drained and sent for bacterial culture. Intravenous injection of ceftriaxone (1 g once daily for seven consecutive days) was administered after the surgery. The patient recovered well on postoperative day 7 and has remained in generally good condition, with good sleeping and eating, normal urination and defecation, and normal body temperature. The results of physical examinations indicated that the patient had stable vital signs. Redness and pain around the abscess were relieved. A small amount of light bloody fluid was found in the drainage catheter upon squeezing the drainage port. The patient was then discharged from the hospital. At 12 weeks after the operation, the patient has recovered from the operation, and she feels clinically well.

### 2.2 Bacterial culture and identification

**Figure 2:**
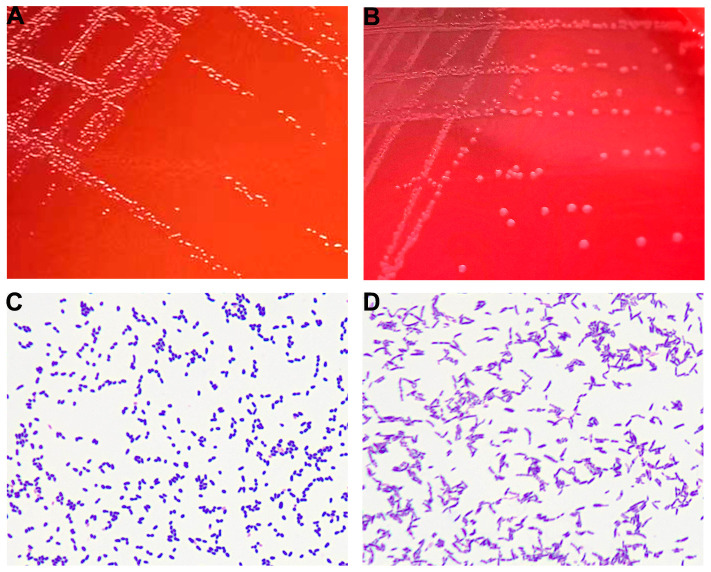
**(A)** Colonies of Isolate A after 48 h of culture on blood agar plate under anaerobic conditions. **(B)** Colonies of Isolate B after 48 h of culture on blood agar plate under anaerobic conditions. **(C)** Gram staining of Isolate A showing gram-positive bacteria appearing as small rods. **(D)** Gram staining of Isolate B showing gram-positive bacteria appearing as rods.

**Figure 3:**
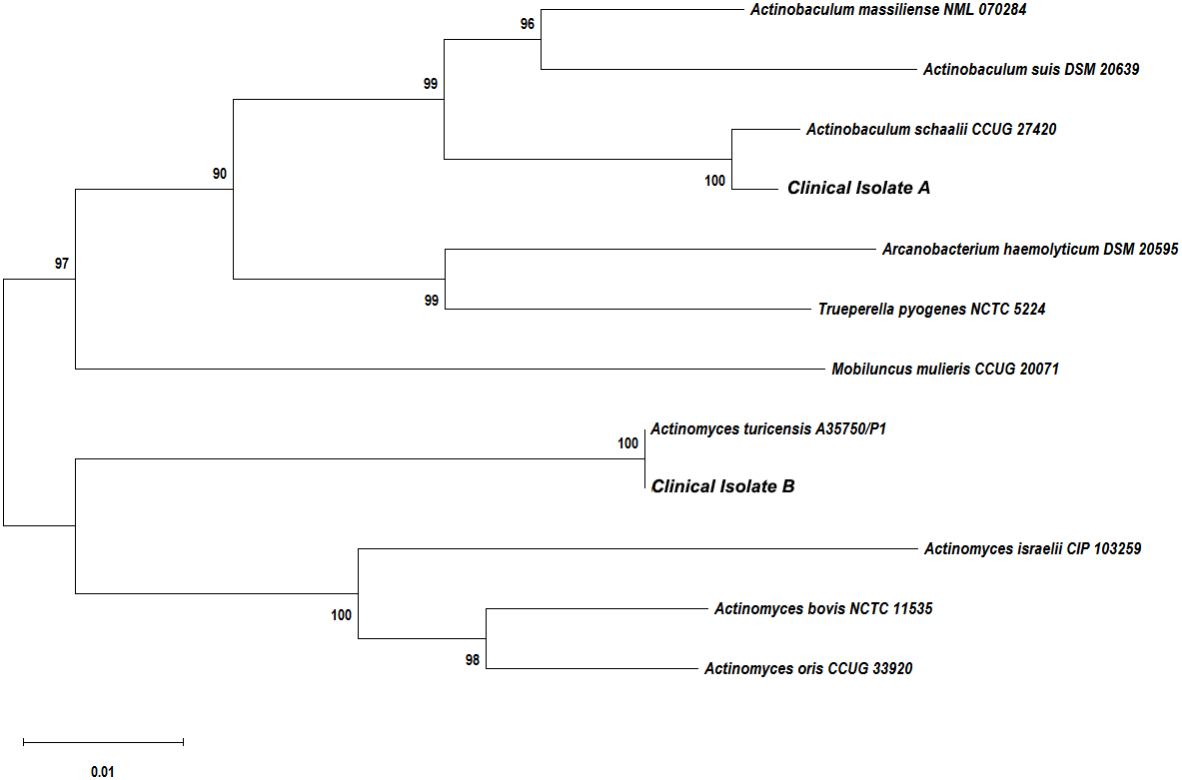
Phylogenetic tree showing the relationships of the patient’s isolates to related species. The neighbor-joining tree was generated with the MEGA11.0 program using maximum composite likelihood method. Bootstrap values were calculated from 1,000 trees. The scale bar indicates the estimated number of substitutions per 50 bases.

The purulent specimen was picked with inoculation loops and inoculated on the Colombian blood agar plates using three quadrant streak method. The plates were placed in different aerobic and anaerobic environments. The regular aerobic culture was placed in a 35°C incubator, and the anerobic culture was placed in a 35°C incubator after air exchange under vacuum in an anerobic tank. Seventy-two hours after incubation, two different types of colonies were visible to naked eye on the agar plates cultured in the anerobic environment, while no colony was observed in the agar plates cultured in the aerobic environment. The two different types of colonies (colonies A and B) from the agar plates cultured anaerobically were isolated and further cultured in an anerobic environment for 48 h. Isolate A colonies were approximately 1 mm in diameter and were round, white, opaque, smooth, raised, and hemolytic (**Figure 2A**). Isolate B colonies were round, gray, opaque, smooth, flat, and moist but not hemolytic (**Figure 2B**). Both types of bacterial colonies were catalase and oxidase negative. Smear staining of both colonies revealed small gram-positive rods in Isolate A (**Figure 2C**) and gram-positive rods in Isolate B (**Figure 2D**). Matrix-assisted laser desorption/ionization time-of-flight mass spectrometry (MALDI Biotyper 3.1, Bruker, Germany) was used to rapidly identify the bacteria using single colonies. It showed that Isolate A colonies was *Actinobaculum schaalii* (identification value: 2.1) and Isolate B colonies were rod-shaped *Actinomyces turicensis* (identification value: 2.2). To further characterize these colonies, 16S rRNA sequencing was performed. Genomic DNAs were extracted using the Ezup Column Bacteria Genomic DNA Purification Kit (Sangon Biotech, China) according to the manufacturer’s instruction, and sequencing libraries were prepared by amplifying the V1–V9 region of the 16S rRNA gene using primers 27F and 1492R by the ABI-3730xL automatic sequencer (Applied Biosystems, USA). The 16S rRNA sequencing (GenBank accession nos. OR294027 and OR294028) analysis and phylogenetic tree analysis (**Figure 3**) further verified that the two bacterial species were *A. schaalii* and *A. turicensis*.

### 2.3 Drug susceptibility testing

Antimicrobial susceptibility testing was performed on Category B medications commonly used in pregnancy, per the United States Food and Drug Administration guidance on drug susceptibility in pregnancy. The minimum inhibitory concentration (MIC) breakpoints for anaerobic bacteria in the Clinical and Laboratory Standards Institute M100-S31 and European Committee on Antimicrobial Susceptibility Testing (Version 10.0) were used as a reference. The E test was performed by inoculating the corresponding bacterial suspensions adjusted to one McFarland concentration in the culture medium and culturing the bacterial samples anaerobically at 35°C. After 48 h of incubation, we determined the MIC of 12 antibiotics, including penicillin, ampicillin, imipenem, ticarcillin/clavulanic acid, erythromycin, clindamycin, piperacillin/tazobactam, amoxicillin/clavulanic acid, ampicillin/sulbactam, metronidazole, cefathiamidine, and ceftriaxone. **Table 1** shows the results of drug susceptibility testing. The two isolates of bacteria were resistant to erythromycin, clindamycin, metronidazole, and imipenem and were susceptible to the remaining eight antibiotics. To treat the abscess in our case, cefathiamidine and ceftriaxone were used. The MIC of *A. turicensis* to cefathiamidine was 0.25, and the MIC of *A. schaalii* to cefathiamidine was 1. The MIC of *A. turicensis* to ceftriaxone was 0.094, and the MIC of *A. schaalii* to ceftriaxone was 0.016. These results indicated that the MICs of both species to ceftriaxone were considerably lower than those to cefathiamidine. This discrepancy in MIC values provides an explanation for the superior efficacy of ceftriaxone over cefathiamidine as a treatment option.

**Table 1:** Drug susceptibility testing of *Actinomyces turicensis* and *Actinobaculum schaalii* strains using the *E* test.

Antibiotics	Actinobaculum schaalii MIC (µg/mL)	Results	Actinomyces turicensis MIC (µg/mL)	Results
Penicillin	0.006	S	0.047	S
Ampicillin	0.125	S	0.125	S
Imipenem	>32	R	>32	R
Ticarcillin/clavulanic acid	0.063	S	0.063	S
Erythromycin	>256	R	>256	R
Clindamycin	>256	R	>256	R
Piperacillin/tazobactam	0.016	S	0.5	S
Amoxicillin/clavulanic acid	0.19	S	0.125	S
Ampicillin/sulbactam	0.047	S	0.094	S
Metronidazole	>256	R	>256	R
Cefathiamidine	1	S	0.25	S
Ceftriaxone	0.016	S	0.094	S

S, susceptible; R, resistant.

## 3 Case series

To conduct a more comprehensive analysis of the prevalence of this infection in skin abscesses, we summarized several cases that were reported to be infected by *A. schaalii* or *A. turicensis* in recent years.

Case 1: A 65-year-old male patient was admitted to the emergency department and was operated on with the diagnosis of Fournier’s gangrene caused by *A. schaalii.* According to the results of drug susceptibility testing, the *A. schaalii* strain was susceptible to penicillin, clindamycin, ciprofloxacin, ceftazidime, and amoxicillin (Tutan et al., 2024).

Case 2 and Case 3: A 66-year-old female patient and a 53-year-old male patient were both diagnosed with a skin abscess. The female patient was coinfected with *A. schaalii* and *Enterobacter cloacae*, whereas the male patient was exclusively infected with *A. schaalii.* Metronidazole was ineffective against the *A. schaalii* isolates, which were susceptible to piperacillin/tazobactam, cefoxitin, imipenem, meropenem, rifampicin, moxifloxacin, chloramphenicol, tetracycline, tigecycline, and vancomycin (Ioannou et al., 2022).

Case 4 and Case 5: Case 4 was an immunocompromised 48-year-old female patient who presented with extensive cellulitis caused by an inguinal abscess. Case 5 was a 25-year-old male patient who presented with a pilonidal abscess. These patients were exclusively infected with *A. schaalii.* The isolate strain of Case 4 was susceptible to penicillin, cloxacillin, amoxicillin/clavulanic acid, imipenem, and clindamycin and resistant to metronidazole and moxifloxacin. In contrast, the strain that infected Case 5 was susceptible to penicillin, cloxacillin, amoxicillin/clavulanic acid, imipenem, and moxifloxacin and resistant to clindamycin and metronidazole. Both patients were treated with cloxacillin and had resolution of the infection (Tena et al., 2014).

Case 6: A 67-year-old Asian male patient without systemic diseases presented to the emergency department and was diagnosed with Fournier’s gangrene caused by *A. turicensis.* The patient underwent radical and repeated debridement and was administered broad-spectrum antibiotics (piperacillin/sulbactam and clindamycin) promptly and exhibited a satisfactory recovery (Mao et al., 2022).

Case 7 and Case 8: A 28-year-old male patient and a 33-year-old male patient were both diagnosed with a skin abscess. The 28-year-old patient was exclusively infected with *A. turicensis*, whereas the 33-year-old patient was coinfected with *A. turicensis* and *Propionibacterium acnes*. The results of drug susceptibility testing indicated that these *A. turicensis* isolates were susceptible to penicillin, clindamycin, ampicillin, cefotaxime, chloramphenicol, erythromycin, rifampin, co-trimoxazole, vancomycin, teicoplanin, and nitrofurantoin; intermediate to teicoplanin; and resistant to metronidazole (Chudácková et al., 2010).

Case 9: This is our case. A 34-year-old pregnant woman was diagnosed with a skin abscess coinfected with *A. schaalii* and *A. turicensis.* These two isolates were resistant to erythromycin, clindamycin, metronidazole, and imipenem and susceptible to penicillin, ampicillin, ticarcillin/clavulanic acid, piperacillin/tazobactam, amoxicillin/clavulanic acid, ampicillin/sulbactam, cefathiamidine, and ceftriaxone. The patient was first treated empirically with 3 days of cefathiamidine, which resulted in no symptom improvement. Subsequently, a surgical procedure involving incision and draining was performed, with the administration of ceftriaxone. The patient exhibited a satisfactory recovery.

## 4 Discussion

Abscesses have the potential to manifest in several types of tissues, with a higher incidence observed in the skin surface, where they can present as either superficial pustules, often referred to as boils, or deep skin abscesses (Daum et al., 2017). In recent years, ultrasonography has emerged as a valuable tool in the diagnosis of skin abscesses owing to its rapidity, noninvasiveness, painlessness, and ability to be readily repeated. More importantly, it is especially useful in differentiating between cellulitis and skin abscesses (Barbic et al., 2017). A systematic review and meta-analysis has revealed that the sensitivity and specificity of ultrasonography in skin abscess diagnosis are 90% and 80%, respectively, which were higher than those of physical examinations (Wu et al., 2022). In this case report, the clinician used color flow Doppler ultrasonography as a diagnostic tool for identifying the skin abscess. This approach offered a higher level of diagnostic confidence in patients who presented with ambiguous signs and symptoms, thereby facilitating prompt initiation of appropriate therapeutic interventions.

Skin abscesses can be caused by bacterial infections, parasites, or foreign substances. Bacterial infections are the most frequent cause. *A. schaalii* is a newly discovered human pathogen that is mostly linked to urinary tract infections (Kakodkar and Hamula, 2022). This meticulous bacterium is not often obtained from skin abscesses. Among the reported skin abscess cases attributed to *A. schaalii*, it has been shown that a significant proportion of these cases exhibit polymicrobial characteristics, with the presence of additional microorganisms, such as *Prevotella* spp., *Fusobacterium* spp., *Arcanobacterium pyogenes*, *Enterococcus faecalis*, and *S. aureus* (Maraki et al., 2017; Kakodkar and Hamula, 2022). However, polymicrobial infection of *A. schaalii* and *A. turicensis* in skin abscesses has not been reported yet.

Infections attributed to *A. schaalii* and *A. turicensis* have the potential to be subject to underdiagnosis. Because of their sluggish rate of proliferation and their resemblance to the indigenous microbial community residing on the skin and mucous membranes, the identification of these entities based on cultural attributes poses challenges (Diallo et al., 2018). With the use of conventional phenotypic tests, such as API system, Rapid ID 32A system, and Rapid ANA II system tests, *A. schaalii* may be misidentified as *Gardnerella vaginalis*, *Arcanobacterium* spp., *Actinomyces meyeri*, or *Actinomyces israelii* (Lotte et al., 2016). A similar dilemma also exists in the identification of *A. turicensis* using conventional laboratory methods. Except for variations in atmospheric conditions, there are limited biochemical assays available for distinguishing between strains of *A. turicensis*, *A. israelii*, and *Actinomyces radingae* (Attar et al., 2007). Molecular techniques, such as the sequencing of the 16S rRNA gene and the utilization of particular real-time PCR, are indeed dependable approaches. However, it is important to note that these procedures can be expensive and may not be easily accessible. The new matrix-assisted laser desorption ionization–time-of-flight identification approach, which is both rapid and cost-effective, has great potential as a reliable tool for accurately identifying *A. schaalii* and *A. turicensis*, as in our case (Ferrand et al., 2016).

The management of *A. schaalii* and *A. turicensis* encompasses a multimodal approach, primarily including the administration of antimicrobial agents and using several surgical interventions that are contingent upon the specific location and underlying cause. However, because of their specific inoculation methods and culture equipment, these anaerobes need more time for cultivation and isolation. Clinicians consistently choose empirical treatment for these bacterial infections (Jin et al., 2020). In the presented case report, the clinician first used cefathiamidine as an empirical therapy approach. However, the efficiency was poor. Subsequently, a procedure including the incision and draining of an abscess was performed, with the administration of ceftriaxone, a third-generation cephalosporin. Following a period of 7 days of medical intervention, the patient exhibited a satisfactory recovery. The antimicrobial susceptibility testing findings can explain the rationale behind the enhanced effectiveness of ceftriaxone compared with cefathiamidine as a therapeutic alternative. Incision and drainage of a skin abscess under either local or general anesthesia is traditionally regarded as the most effective therapy for these abscesses (Rühle et al., 2021). However, some clinicians have indicated that intravenous antibiotic therapy alone, without surgical surgery, is adequate in certain abscesses (Ketterer et al., 2023). In our case, because of the relatively large size of the skin abscess, our clinician performed incision and drainage along with intravenous antibiotic therapy. Finally, the patient exhibited a satisfactory recovery.

Antimicrobial susceptibility testing of *A. schaalii* and *A. turicensis* is seldom performed in clinical microbiology laboratories mostly because of the prevailing assumption that strains of *A. schaalii* and *A. turicensis* are often sensitive to β-lactam antibiotics (Barberis et al., 2017). Consistent with the findings of previous researchers (Steininger and Willinger, 2016; Barberis et al., 2017), the present investigation revealed that both of the isolates under examination demonstrated susceptibility to most β-lactams, except for their resistance to imipenem. In addition, it is important to acknowledge that several reported strains of *A. schaalii* and *A. turicensis* have exhibited susceptibility to clindamycin, erythromycin, and metronidazole (Steininger and Willinger, 2016; Barberis et al., 2017). In contrast, the results of our study demonstrate that the isolated *A. schaalii* and *A. turicensis* strains exhibited resistance to the aforementioned three antibiotics.

One limitation in our case report is the presence of a microbial infection that lacks a discernible etiology. The most prevalent risk factors for these infections include advanced age, urologic-related predisposing diseases, and immunosuppressive illnesses (Lotte et al., 2016; Khan et al., 2022). However, none of these identified risk factors align with the characteristics exhibited by our patient.

## 5 Conclusion

*A. schaalii* and *A. turicensis* have been observed as emerging pathogens with clinical manifestations that extend beyond urinary tract infections. Nevertheless, the clinical importance of these bacterial infections continues to be underestimated. Despite the persistent challenges in culturing and isolating these bacteria, it is imperative for clinicians to proactively request susceptibility testing for these pathogens.

## Data availability statement

The datasets presented in this study can be found in online repositories. The names of the repository/repositories and accession number(s) can be found in the article/supplementary material.

## Ethics statement

The studies involving humans were approved by Ethics Committee of Jinan Maternity and Child Care Hospital. The studies were conducted in accordance with the local legislation and institutional requirements. The participants provided their written informed consent to participate in this study. Written informed consent was obtained from the individual(s) for the publication of any potentially identifiable images or data included in this article.

## Author contributions

PL: Funding acquisition, Writing – original draft, Writing – review & editing, Formal analysis. KS: Data curation, Writing – original draft. RL: Data curation, Writing – original draft. XC: Funding acquisition, Writing – review & editing.
